# Roles and Regulation of Ketogenesis in Cultured Astroglia and Neurons Under Hypoxia and Hypoglycemia

**DOI:** 10.1177/1759091414550997

**Published:** 2014-09-10

**Authors:** Shinichi Takahashi, Takuya Iizumi, Kyoko Mashima, Takato Abe, Norihiro Suzuki

**Affiliations:** 1 Department of Neurology, Keio University School of Medicine, Tokyo, Japan

**Keywords:** acetoacetate, AMP-activated protein kinase, β-hydroxybutyrate, ketone bodies, long-chain fatty acids, palmitic acid

## Abstract

Exogenous ketone bodies (KBs), acetoacetate (AA), and β-hydroxybutyrate (BHB) act as alternative energy substrates in neural cells under starvation. The present study examined the endogenous ketogenic capacity of astroglia under hypoxia with/without glucose and the possible roles of KBs in neuronal energy metabolism. Cultured neurons and astroglia were prepared from Sprague-Dawley rats. Palmitic acid (PAL) and l-carnitine (LC) were added to the assay medium. The 4- to 24-hr production of AA and BHB was measured using the cyclic thio-NADH method. ^14^C-labeled acid-soluble products (KBs) and ^14^CO_2_ produced from [1-^14^C]PAL were also measured. l-[U-^14^C]lactic acid ([^14^C]LAC), [1-^14^C]pyruvic acid ([^14^C]PYR), or β-[1-^14^C]hydroxybutyric acid ([^14^C]BHB) was used to compare the oxidative metabolism of the glycolysis end products with that of the KBs. Some cells were placed in a hypoxic chamber (1% O_2_). PAL and LC induced a higher production of KBs in astroglia than in neurons, while the CO_2_ production from PAL was less than 5% of the KB production in both astroglia and neurons. KB production in astroglia was augmented by the AMP-activated protein kinase activators, AICAR and metformin, as well as hypoxia with/without glucose. Neuronal KB production increased under hypoxia in the absence of PAL and LC. In neurons, [^14^C]LAC and [^14^C]PYR oxidation decreased after 24 hr of hypoxia, while [^14^C]BHB oxidation was preserved. Astroglia responds to ischemia *in vitro* by enhancing KB production, and astroglia-produced KBs derived from fatty acid might serve as a neuronal energy substrate for the tricarboxylic acid cycle instead of lactate, as pyruvate dehydrogenase is susceptible to ischemia.

## Introduction

Adult brain function is exclusively dependent on the oxidative metabolism of glucose under normal physiological conditions (Clarke and Sokoloff, 1999; Dienel, 2009), while ketone bodies (KBs), which are mainly synthesized and supplied by the liver (i.e., exogenous KBs; Robinson and Williamson, 1980; Guzmán and Geelen, 1993), can fuel the infantile brain (Edmond et al., 1985; Vannucci and Simpson, 2003). Even the adult brain can utilize exogenous KBs when glucose availability is limited (Clarke and Sokoloff, 1999; Dienel, 2009), such as during starvation, prolonged hypoglycemia, or because of insulin resistance associated with diabetes mellitus (Nehlig, 2004; Veech, 2004). Moreover, infusions of [2,4-^13^C_2_]-β-hydroxybutyrate and ^1^H–^13^C polarization transfer spectroscopy in normal human subjects revealed the entry and metabolism in the nonfasted brain (Pan et al., 2002). Furthermore, experimental evidence suggests that exogenous KBs exert neuroprotective roles under several circumstances, including cerebral ischemia (Prins, 2008; Puchowicz et al., 2008). In contrast to exogenous KBs, however, the roles and regulation of endogenous KBs remain to be determined.

Fatty acids serve as a main energy substrate of cardiac myocytes, and skeletal muscle displays substantial metabolic flexibility, with the capacity to switch from predominantly lipid oxidation and high rates of fatty acid uptake during fasting conditions to the suppression of lipid oxidation and increased glucose uptake, oxidation, and storage under insulin-stimulated conditions (Andres et al., 1956; Kelley et al., 1990). Although acetyl-CoA, an end product of the β-oxidation of fatty acids, enters the tricarboxylic acid (TCA) cycle in the mitochondria of these cells, neural cells prefer glucose-derived acetyl-CoA to fatty acid-derived acetyl-CoA. Therefore, fatty acid-derived acetyl-CoA produced in neural cells by β-oxidation, the amount of which is minimal (if any), leads to ketogenesis (acetoacetate [AA] and β-hydroxybutyrate [BHB]).

Fatty acids are bound to albumin in the blood, and only free fatty acids can cross the blood–brain barrier (BBB; Dhopeshwarkar and Mead, 1973; Smith and Nagura, 2001). Although the brain levels of soluble fatty acids have not been reported, fatty acids have long been known to be BBB permeable. Neural cells also reportedly express fatty acid-binding proteins that take up free fatty acids (Mitchell et al., 2011). Long-chain fatty acids (>12 carbons) such as palmitic acid (PAL, 16 carbons), a predominant fatty acid in the body, are then metabolized to produce long-chain fatty acid acyl-CoA. Fatty acid acyl-CoA is then transported into mitochondria by carnitine palmitoyltransferase I (CPT-I), which exists in the outer membrane of mitochondria and undergoes β-oxidation. Because astrocytes envelop microvessels in the brain (Mathiisen et al., 2010), they are likely to be the main site of fatty acid metabolism in the brain.

Although only astrocytes are thought to be able to utilize fatty acids for CO_2_ production (Edmond et al., 1987 ;Auestad et al., 1991), not only astrocytes but other kinds of brain cells also potentially utilize KBs (Edmond et al., 1987; Auestad et al., 1991; McKenna et al., 1993; McKenna, 2012). A limited number of studies performed by essentially a single laboratory have revealed that endogenous KBs produced from fatty acid by astrocytes may support neuronal energy metabolism in place of glucose or lactate (Blázquez et al., 1998, 1999; Guzmán and Blázquez, 2001, 2004). To date, however, whether hypoxia or hypoglycemia enhances astroglial KB synthesis has not been extensively studied (Taïb et al., 2013). Furthermore, neuronal utilization of astrocyte-derived KBs after hypoxia or hypoglycemia remains to be determined.

Guzmán and Blázquez (2001) reported that AMP-activated protein kinase (AMPK) regulates astroglial ketogenesis by phosphorylating acetyl-CoA carboxylase (ACC), thereby inhibiting ACC activity and reducing cytosolic malonyl-CoA—a major physiological inhibitor of CPT-I (a rate-limiting enzyme of fatty acid metabolism). In fact, 5-amino-1 -β-d-ribofuranosyl-imidazole-4-carboxamide (AICAR), a cell-permeable analog of AMP that activates AMPK (Corton et al., 1995), enhances ketogenesis in astroglia. Because AMPK is a sensor of AMP/ATP, an indicator of the energy reserve in cells, Guzmán and Blázquez speculated that hypoxia/ischemia may stimulate astroglial ketogenesis and proved that chemical hypoxia induced by 1 mmol/L of NaN_3_, which inhibits cytochrome oxidase and thus the mitochondrial respiratory chain (Leary et al., 2002), for 1 hr did indeed enhance ketogenesis in cultured astroglia (Blázquez et al., 1999).

Hypoxia also activates glycolytic activity in both astroglia and neurons, leading to enhanced lactate production. After reoxygenation, lactate can fuel neuronal oxidative metabolism if the function of the mitochondrial pyruvate dehydrogenase (PDH) enzyme complex (PDHC) is preserved. However, PDH is known to be susceptible to hypoxia or reactive oxygen species (ROS) induced by reoxygenation (Cardell et al., 1989; Fukuchi et al., 1998). Therefore, lactate may not be a suitable substrate for neuronal metabolism after ischemia–reperfusion. In contrast, KBs, especially BHB, can enter the TCA cycle directly in the absence of PDHC. The present study examined the effects of hypoxia/hypoglycemia and the pharmacological activation of AMPK by AICAR as well as 1,1-dimethylbiguanide hydrochloride (metformin; Fulgencio et al., 2001; Zang et al., 2008) on ketogenesis in cultured neurons and astroglia and compared the neuronal oxidation of lactate/pyruvate with that of BHB.

## Materials and Methods

### Animals

Timed-pregnant Sprague-Dawley rats were purchased from Japan SLC, Inc. (Hamamatsu, Japan). All the animal procedures were performed in accordance with “The Animal Experimentation Guidelines” of Keio University School of Medicine and were approved by the Committee on Animal Care and Use, Keio University.

### Chemicals

Chemicals and materials were obtained from the following sources: [1-^14^C]palmitic acid ([^14^C]PAL; specific activity, 2.22 GBq/mmol), l-[U-^14^C]lactic acid ([^14^C]LAC; specific activity, 6.21 GBq/mmol), [1-^14^C]pyruvic acid ([^14^C]PYR; specific activity, 352 MBq/mmol), Insta-Fluor Plus, Insta-Gel Plus, and hyamine hydroxide 10-X were obtained from Perkin-Elmer Life Sciences (Boston, MA, USA); β-[1-^14^C]hydroxybutyric acid ([^14^C]BHB; specific activity, 2.035 GBq/mmol) was obtained from American Radiolabeled Chemicals, Inc. (St. Louis, MO, USA); Dulbecco’s modified Eagle media (DMEM) with or without glucose, penicillin, and streptomycin were obtained from Life Technologies (Grand Island, NY, USA); defined fetal bovine serum (FBS) was obtained from HyClone Laboratories (Logan, UT, USA); and all other chemicals were obtained from Sigma (St. Louis, MO, USA).

### Preparation of Cells

The primary astroglial cultures were prepared from the cerebral cortex of rat pups 24 to 48 hr after birth (Takahashi et al., 2012a). The mechanically dissociated cells from the cortex (2.5 × 10^5^ cells/ml) were plated (15 ml/flask) in uncoated 75-cm^2^ culture flasks (Sumitomo Bakelite, Tokyo, Japan) and cultured in a glucose-containing medium (final concentration, 12 mmol/L of d-glucose) comprising DMEM with 10% (v/v) FBS, penicillin (100 U/ml), and streptomycin (100 µg/ml) at 37℃ in humidified air containing 21% O_2_ and 7% CO_2_ (Day 0). The culture medium was changed every 2 days until the cultures reached confluence. On Day 11, the adherent cells were treated with trypsin-EDTA solution, suspended in fresh high (12 mmol/L)-glucose medium, and placed in uncoated 12-well culture plates (0.8 ml/well, respectively; Nalge Nunc., Rochester, NY, USA) or in 25-cm^2^ culture flasks (5 ml/flask; Nalge Nunc). From the day after subculturing, the cells were cultured in DMEM, FBS, penicillin, and streptomycin and a final d-glucose concentration of 12 mmol/L for 10 days. The culture medium was changed twice a week, and the cells (secondary astroglial cultures) were used for assays once they reached confluence (on Day 21 *in vitro*). Because the rates of astroglial glucose consumption are very high, the nutrient medium had to be replaced with fresh medium containing 12 mmol/L of d-glucose every 3 or 4 days. In fact, the glucose concentration in the medium fell to 5 to 7 mmol/L 4 days after medium change (data not shown). If the nutrient medium remained untouched for more than 5 days, the glucose concentration fell to zero (data not shown).

The primary neuronal cultures were prepared from the cortex and striatum of fetal rats on embryonic Day 16, as described previously (Takahashi et al., 2012a). The mechanically dissociated cells were placed in 12-well culture plates (0.8 ml/well, respectively) or 25-cm^2^ culture flasks (5 ml/flask) coated with poly-l-lysine (5 µg/ml). For the neuronal cultures, viable cells (1.5 × 10^6^ cells per ml) that excluded Trypan blue were placed in the cultures, and cytosine arabinoside (10 µmol/L) was added 72 hr later to induce the mitotic arrest of the astroglia. The cells were cultured in a glucose medium (final concentration, 12 mmol/L d-glucose) at 37℃ in humidified air containing 21% O_2_ and 7% CO_2_. *In vitro* assays were performed using cultures that were 7 or 8 days old. Because neurons do not tolerate medium change and replacement by fresh nutrient medium severely damages them (Driscoll et al., 1993), the nutrient medium remained untouched until the experiments were initiated. At the time of experiment (usually on Day 7), the glucose concentration was approximately 5 to 7 mmol/L (data not shown), which is close to astroglial glucose condition.

### Preparation of the Assay Solution for PAL Oxidation and KB Synthesis

For the measurement of [^14^C]PAL oxidation to ^14^CO_2_ and KB synthesis, the nutrient medium was replaced with assay solution containing PAL (100 µmol/L) acid and l-carnitine (LC; 1 mmol/L) with the appropriate drugs.

On the day of use, PAL stock solutions of 200 mmol/L were prepared in 100% ethanol (EtOH). Working water-soluble solutions of 5 mmol/L of PAL with or without 0.04 mmol/L [^14^C]PAL were then generated by incubating the PAL in phosphate-buffered saline (PBS) containing 10% endotoxin-free and fatty acid**-**free bovine serum albumin (BSA) at 37℃ for 30 to 60 min with occasional vortexing. This solution was then added to the assay solutions (20 µL/ml of Dulbecco’s balanced salt solution [DBSS] containing 110 mmol/L NaCl, 5.4 mmol/L KCl, 1.8 mmol/L CaCl_2_, 0.8 mmol/L MgSO_4_, 0.9 mmol/L NaH_2_PO_4_, and 44 mmol/L NaHCO_3_ containing 2 mmol/L of d-glucose for the ^14^C method or 20 µL/ml of nutrient medium, DBSS, or DMEM for the cyclic thio-NADH method). Equal volumes of the PBS /EtOH /10% fatty acid-free BSA vehicle were applied to the control cells.

When 24-hr hypoxia experiment was performed, nutrient medium was used. For neuronal cells, nutrient medium in which cells had been grown was collected and used for the assay by adding PAL and LC because replacement by fresh medium severely damages neurons after 24 hr (Driscoll et al., 1993). For astroglia, fresh nutrient medium that was used for medium change every 3 or 4 days during cultivation was used for the assay by adding PAL and LC. When combination study of hypoxia and hypoglycemia was performed, we used DBSS or DMEM without glucose for shorter period of time (4 or 12 hr) that both neuronal and astroglial cells can tolerate.

### Measurement of AA and BHB Using the Cyclic Thio-NADH Method

The rates of production of two major KBs (i.e., AA and BHB) were measured using the cyclic thio-NADH method (Laun et al., 2001). After the cells were incubated with 0.5 ml of the assay solution containing 100 µmol/L PAL and 1 mmol/L LC, 300 µL of the supernatant was sampled and used. First, 150 µL of 300 µL supernatant was used to determine total KBs (BHB and AA) content. By the addition of BHB dehydrogenase and β-nicotinamide adenine dinucleotide disodium (NADH + H^+^, reduced form), AA was converted to BHB. Then, thio-NAD^+^, the oxidized form of β-thionlactinamide adenine dinucleotide, was added to convert thio-NAD^+^ to thio-NADH. The produced thio-NADH was quantified using spectrophotometry (404 nm) to determine total BHB (i.e., total KBs) content in 150 µL of supernatant. Second, the remaining 150 µL of 300 µL supernatant was used to determine BHB content. AA was removed by converting AA to acetone and CO_2_ through the addition of AA dehydrogenase. Then, BHB was quantified by converting to AA by adding thio-NAD^+^, as described earlier. Finally, the AA content was calculated based on the difference between the total KB (the first sample) and the BHB (the second sample). Net production (negative values indicate consumption) of KBs after treatment was calculated by subtracting the baseline value of KB contents and standardized by cellular protein (µg) and expressed by per assay period (min or hr).

### Measurement of the Rate of [^14^C]PAL Oxidation to ^14^CO_2_

The rate of PAL oxidation (i.e., the oxidative metabolism of PAL in the TCA cycle) was measured using a modification of the method described by Guzmán and Blázquez (2001). After cells cultured in 25-cm^2^ culture flasks were washed twice with PBS without Ca^2+^ and Mg^2+^ containing no glucose, DBSS containing 2 mmol/L of d-glucose, 100 µmol/L PAL, 0.2% BSA, and 1 mmol/L LC was added. These solutions were labeled using [^14^C]PAL (original concentrations: 3.7 MBq/ml, 1.67 mmol/L) to obtain a final concentration of 0.84 µmol/L. The flasks were capped with rubber stoppers containing a center well and were incubated at 37℃ for 60 min. The ^14^CO_2_ produced was trapped using a cotton ball placed in the center well containing 100 µL of hyamine hydroxide 10-X. The reactions were terminated by the injection of 250 µL of HClO_4_ (2 mol/L) through the rubber stopper, and the flasks were kept at 4℃ overnight to trap the ^14^CO_2_. The center wells were then transferred to 20**-**ml glass scintillation counter vials, and 500 µL of EtOH and 10 ml of Insta-Fluor Plus were added. The ^14^C contents of the vials were evaluated using a liquid scintillation counter. Waniewski and Martin (2004) reported that substantial ^14^C counts were obtained from a flask without cells. Therefore, the ^14^C count obtained for a flask without cells, in which the reaction had been stopped at 60 min, was regarded as the background value and was subtracted from the values of the flasks containing cells in the present study.

### Measurement of the Rate of KB Synthesis From [^14^C]PAL

The [^14^C]PAL assay was initiated by the addition of 0.84 µmol/L (final concentration) of albumin-bound [^14^C]PAL, PAL (100 µmol/L), 0.2% BSA, LC (1 mmol/L), and 2 mmol/L of d-glucose to cultured cells, as described earlier. The reaction was stopped with 250 µL of HClO_4_ (2 mol/L) after 60 min (PAL oxidation assay). After the overnight collection of ^14^CO_2_, KBs were extracted and quantified exactly as described by Guzmán and Blázquez (2001). The assay solution was collected and spun down (1,500 × g for 5 min), and 50 µL of supernatant was transferred to a 20**-**ml glass scintillation counter vial containing 1 ml of H_2_O; 10 ml of Insta-Gel Plus was then added. The ^14^C contents of the vials were evaluated using a liquid scintillation counter. As for KB production, background values were determined using blank flask without cells, as described earlier.

The cell carpets remaining in the incubation flasks after the removal of the reaction mixtures were then digested with 5 ml of 0.1 mol/L NaOH, and their protein contents were determined. The rates of total PAL metabolism (pmol PAL/µg protein/60 min) based on the conversion from [^14^C]PAL to ^14^CO_2_ or the acid-soluble fraction over 60 min were measured.

### Measurement of the Rates of [^14^C]LAC, [^14^C]PYR, and [^14^C]BHB Oxidation to ^14^CO_2_

The rates of [^14^C]LAC, [^14^C]PYR, and [^14^C]BHB oxidation to ^14^CO_2_ were measured using a modification of a previously described method (Abe et al., 2006). The assay procedure was basically the same as the one used for the measurement of the rate of [^14^C]PAL oxidation to ^14^CO_2_ described earlier. The assay solutions that contained no glucose consisted of 2.5 ml of DBSS containing 2 mmol/L of LAC, 2 mmol/L of PYR, or 4 mmol/L of d-/l-BHB with the appropriate ^14^C-labeled tracers. These solutions were labeled by adding 1.0 µL/ml of [^14^C]LAC (original concentrations: 3.7 MBq/ml, 0.60 mmol/L), [^14^C]PYR (original concentrations: 3.7 MBq/ml, 10.51 mmol/L), or [^14^C]BHB (original concentrations: 3.7 MBq/ml, 1.8 mmol/L), respectively. For each substrate, ^14^C count as the background value was obtained from a flask without cells. Cellular oxidative capacity of the LAC, PYR, or BHB was calculated by subtracting the background value of each substrate and standardized by protein contents.

### Experimental Protocol

To assess the effect of hypoxia on ketogenesis, cells were placed in a hypoxic chamber (Multi-gas incubator APM-30DR; ASTEC, Fukuoka-ken, Japan) at 37℃ in humidified air containing 1% O_2_ and 7% CO_2_ for 4, 12, or 24 hr with or without glucose. When pharmacological activators of AMPK (AICAR or metformin), cilostazol, rotenone, or glutamate were applied, the appropriate drugs in a suitable vehicles, that is, 0.1% (v/v) DMSO for cilostazol, 0.1% (v/v) EtOH for rotenone, and water 0.1% (v/v) for glutamate, were added to the medium, and the cells were incubated for 24 hr. For the neuronal cultures, as changes in the medium *per se* can damage the cells through glutamate toxicity (Driscoll et al., 1993), the drugs were added to the culture media in which the cells had been grown to achieve the final concentrations. For the assays carried out with glucose-free condition, DBSS or DMEM without glucose was used instead of the nutrient medium for both neurons and astroglia by eliminating FBS. After 4 to 24 hr of exposure, 0.3 ml of the culture medium supernatant was sampled and used for the AA and BHB measurements. The cell carpet was lysed using 0.1 mol/L of NaOH, and the protein contents were measured using a bicinchoninic acid (BCA) assay (Smith et al., 1985).

For the glucose-deprivation assays and the [^14^C]assays, the nutrient medium was removed, and the cells were washed twice with PBS containing no glucose. Then, the cells were incubated with DBSS and tracer doses of [^14^C]PAL (with 2 mM glucose), or with [^14^C]LAC, [^14^C]PYR, or [^14^C]BHB (all in the absence of glucose).

### Statistical Analyses

Statistical comparisons of the values obtained for each group were performed using grouped *t* tests or a one**-**way analysis of variance (ANOVA) followed by the Dunnett test for multiple comparisons. A *p* value of <.05 was considered statistically significant.

## Results

### Fatty Acid Metabolism in Neurons and Astroglia as Assessed Using [^14^C]PAL

**Figure 1.**
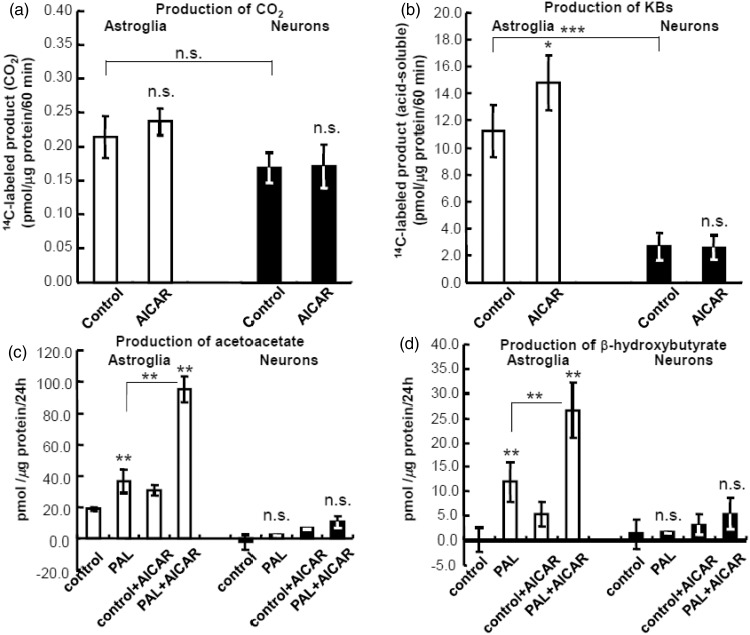
Fatty acid oxidation and ketogenesis by neurons and astroglia. (a) Fatty acid oxidation in neurons (filled bars) and astroglia (blank bars) as determined by quantifying the amount of ^14^CO_2_ derived from [1-^14^C]palmitic acid ([^14^C]PAL). 5-Amino-1 -β-d-ribofuranosyl-imidazole-4-carboxamide (AICAR), an AMP-activated protein kinase (AMPK) activator, did not alter the oxidative metabolism. (b) Total ketone body production in neurons and astroglia as determined by collecting the acid-soluble fraction derived from [^14^C]PAL. AICAR enhanced astroglial KB production. (c) Production of acetoacetate (AA) from PAL in neurons and astroglia as determined using the cyclic thio-NADH method. (d) Production of β-hydroxybutyrate (BHB) from PAL in neurons and astroglia as determined using the cyclic thio-NADH method with palmitic acid (PAL) and l-carnitine (LC). Glucose (2 mmol/L) was included in all assay media. Both AA and BHB production in astroglia were enhanced by AICAR, while neuronal AA or BHB was not statistically significantly altered by AICAR. Values are the mean ± SD (*n* = 4 for a and b, *n* = 6 for c and d). n.s. = not significant. *p < .05; **p < .05; ***p < .001 (grouped *t* test).

The rates of fatty acid (PAL) oxidation in the presence of glucose in both neurons and astroglia (Figure 1(a)) were similar and much smaller (approximately 1%–5%) than those of KB production, which were predominant in both cell types (Figure 1(b)), and of glucose oxidation (not shown). According to our previous report, the rate of neuronal [U-^14^C]glucose oxidation is approximately 12 pmol/µg protein/60 min and that of astroglial oxidation is 2 to 6 pmol/µg protein/60 min (Abe et al., 2006). Interestingly, in contrast to glucose oxidation, which is much higher in neurons than in astroglia, the rates of PAL oxidation were similar in both cell types. The rates of CO_2_ production from [^14^C]PAL were 0.17 and 0.22 pmol/µg protein/60 min in neurons and astroglia (Figure 1(a); *p* = .054), respectively. In contrast, KB production from PAL was about 5-fold higher in astroglia than in neurons (Figure 1(b); *p* < .001). AICAR (an AMPK activator) did not alter PAL oxidation by neurons and astroglia, whereas it enhanced astroglial KB production (*p* < .05, grouped *t* test) but did not alter neuronal KB production.

### KB Production by Neurons and Astroglia in the Presence or Absence of PAL and LC

The levels of KBs (AA and BHB) derived from fatty acid (PAL) were 5 to 10 times higher in astroglia than in neurons (Figure 1(c) and (d)). In both types of cells, KB production was not completely PAL-dependent, as astroglia and neurons both produced KBs in the absence of PAL. In astroglia, AA accounted for the major KB fraction (Figure 1(c)), compared with BHB (Figure 1(d)). The astroglial productions of both AA and BHB were augmented by AICAR, while the neuronal KB production was not affected by AICAR (Figure 1(c) and (d)).

### Effects of Metformin and Cilostazol on Ketogenesis in Astroglia and Neurons

**Figure 2.**
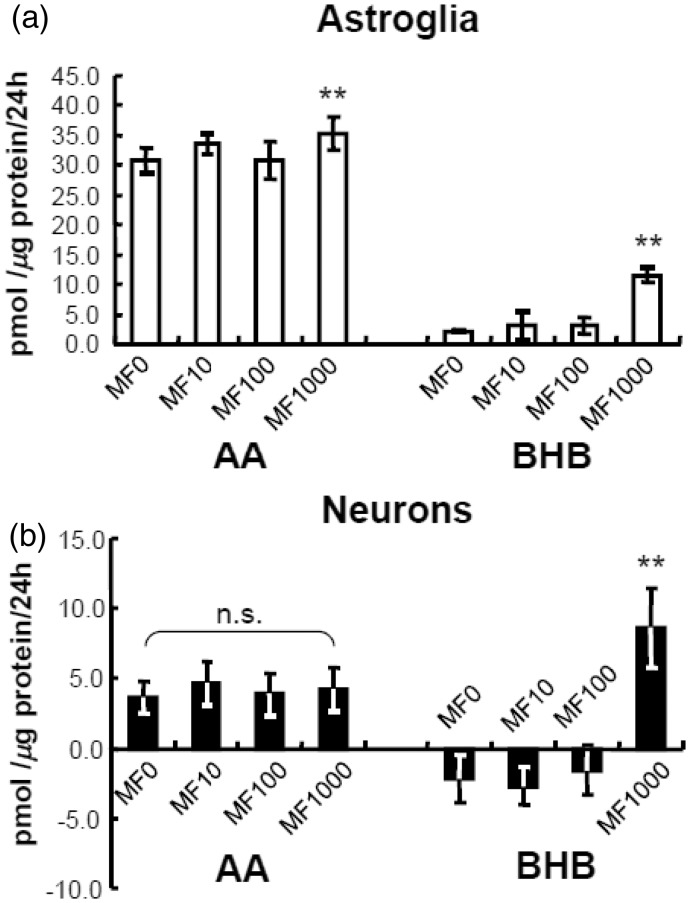
Effects of metformin (MF) on ketogenesis from palmitic acid by neurons and astroglia. Metformin (1,000 µmol/L) activated the production of acetoacetate (AA) and β-hydroxybutyrate (BHB) in astroglia (blank bars, a) and neurons (filled bars, b) as determined using the cyclic thio-NADH method during 24 hr in the presence of palmitic acid (PAL) and l-carnitine (LC). Glucose (2 mmol/L) was included in all assays. Note that the neuronal nutrient medium that had been present for 5 to 6 days was collected and reused for the neuronal KB assays, and negative values indicate net consumption of KBs that were present in the 5- to 6-day medium during the subsequent assay interval. Neurons consumed small amounts of BHB during the experimental interval. Astrocytes are not sensitive to medium change, and fresh medium was used for the astrocyte assays. Values are the mean ± SD (*n* = 6). n.s. = not significant. **p < .01 (Dunnett test for multiple comparisons following ANOVA).

We also examined the pharmacological activation of AMPK by metformin (1–1,000 µmol/L) on ketogenesis in cultured neurons and astroglia. Metformin is another AMPK activator that is used clinically for the treatment of diabetes mellitus. Lower concentrations of metformin (1–100 µmol/L) did not alter KB production by astroglia or neurons, whereas a higher concentration (1,000 µmol/L) did, indeed, induce KB (BHB) production in astroglia (Figure 2(a)) and neurons (Figure 2(b)). Neuron consumed small amounts of BHB during the experimental interval (negative values, Figure 2(b)). Notably, AICAR is a stronger inducer of ketogenesis in astroglia than metformin (compare Figure 1(c) and (d) with Figure 2(a)). Although the metformin concentration used for the treatment of diabetic patients is approximately 10 µmol/L, this concentration did not affect KB production in both cell types. In contrast, metformin at a concentration of 1,000 µmol/L reportedly activates AMPK *in vitro* (Zang et al., 2008; Figure 2); thus, the clinical relevance of this concentration remains to be established.

**Figure 3.**
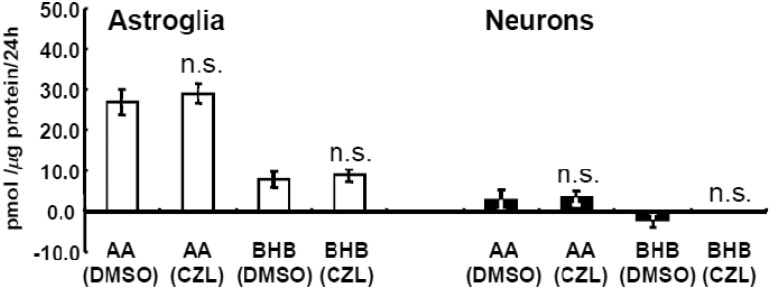
Effects of cilostazol (CZL) on ketogenesis from palmitic acid by neurons and astroglia. Effects of 3 µmol/L of CZL on production of acetoacetate (AA) and β-hydroxybutyrate (BHB) in neurons (filled bars) and astroglia (blank bars) as determined using the cyclic thio-NADH method during 24 hr in the presence of palmitic acid (PAL) and l-carnitine (LC). Glucose (2 mmol/L) was included in all assays. Values are the mean ± SD (*n* = 6 for a and b). n.s. = not significant (grouped *t* test).

In addition to AMPK, cAMP-dependent protein kinase (PKA) has been known to phosphorylate and to inhibit ACC, thus leading to ketogenesis (Blázquez et al., 1998). Therefore, we examined the effects of cilostazol, a potent inhibitor of type 3 phosphodiesterase (PDE3) that induces the activation of PKA through an elevation of cAMP (Thompson et al., 2007), on ketogenesis in neural cells. When administered at clinically relevant concentrations, cilostazol (a PKA activator), which may inhibit ACC through phosphorylation and enhance CPT-I by reducing malonyl-CoA, did not affect KB production in neurons or astroglia (Figure 3).

### Hypoxia-Enhanced Astroglial Ketogenesis and the Enhancement of Neuronal BHB Production Were Not Completely PAL Dependent

**Figure 4.**
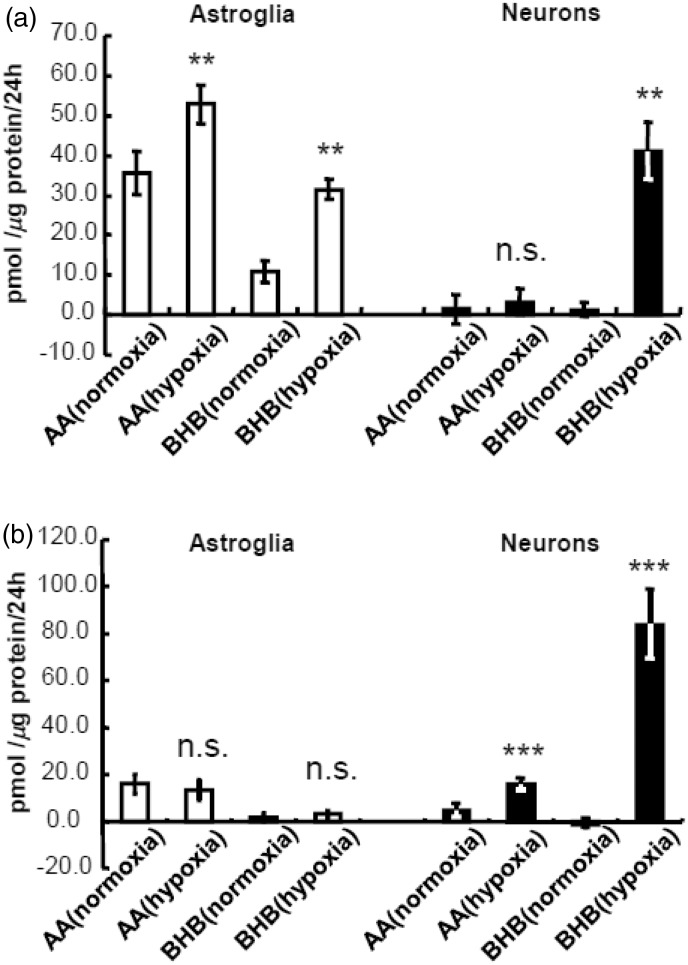
Effects of hypoxia on ketogenesis in the presence and absence of palmitic acid by neurons and astroglia. (a) Production of acetoacetate (AA) and β-hydroxybutyrate (BHB) in neurons (filled bars) and astroglia (blank bars) as determined using the cyclic thio-NADH method during 24 hr under normoxia (21% O_2_) or hypoxia (1% O_2_) in the presence of palmitic acid (PAL) and l-carnitine (LC). Hypoxia increased AA and BHB production in astroglia but only increased BHB production in neurons. (b) Production of AA and BHB in neurons and astroglia under hypoxia in the absence of PAL and LC. Glucose (2 mmol/L) was included in all assays. Astroglial AA and BHB production were markedly reduced by the elimination of PAL and LC, and the hypoxia-induced augmentation disappeared. In contrast, the hypoxia-induced enhancement of BHB production was preserved in neurons even in the absence of PAL and LC. Values are the mean ± SD (*n* = 6 for a and b). n.s. = not significant. **p < .01; ***p < .001 (grouped *t* test).

Hypoxia (1% O_2_ for 24 hr) augmented the astroglial production of both AA and BHB, as expected (Figure 4(a)). Unexpectedly, however, neuronal BHB production, but not AA production, also increased under hypoxia (Figure 4(a)). The enhancement of astroglial KB production under hypoxia disappeared with the elimination of PAL (Figure 4(b)), indicating that hypoxia does not enhance the influx of glycolytic products available for KB generation. In contrast, the enhanced production of BHB by neurons under hypoxia was not affected in the absence of PAL (Figure 4(b)). Moreover, an increase in the mitochondrial redox state (NADH/NAD^+^) might facilitate the conversion of AA to BHB as well as AA production *per se*.

### Combination of Hypoxia and Hypoglycemia Enhances Astroglial KB Synthesis

**Figure 5.**
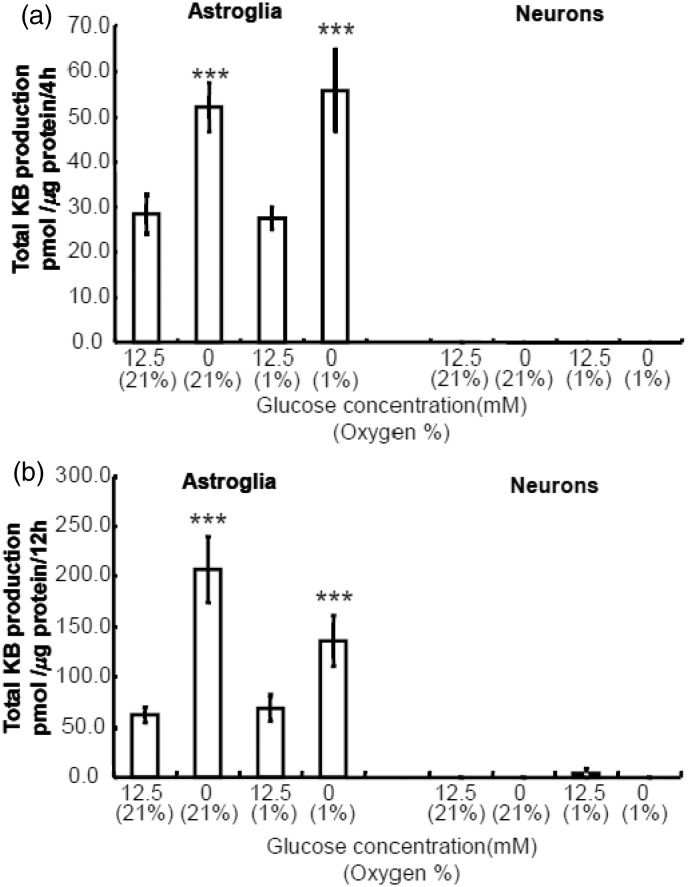
Effects of hypoxia with or without glucose-deprivation on ketogenesis from exogenous palmitic acid by neurons and astroglia. The effects of hypoxia (1% O_2_) with glucose (12.5 mmol/L) or without glucose for 4 hr (a) and 12 hr (b) on neuronal or astroglial total ketone body (KB) production were examined using Dulbecco’s balanced salt solution (DBSS) containing 110 mmol/L NaCl, 5.4 mmol/L KCl, 1.8 mmol/L CaCl_2_, 0.8 mmol/L MgSO_4_, 0.9 mmol/L NaH_2_PO_4_, and 44 mmol/L NaHCO_3_. Neither neurons nor astroglia tolerate hypoxia without glucose (i.e., oxygen–glucose deprivation, OGD) for 24 hr. Neuronal KB productions during 4 or 12 hr were below the measurable concentration by the present assay method. Hypoxia with glucose for 4 or12 hr did not elicit statistically significant increases in KB production by astroglia. Values are the mean ± SD (*n* = 6). ***p < .001 (grouped *t* test).

**Figure 6.**
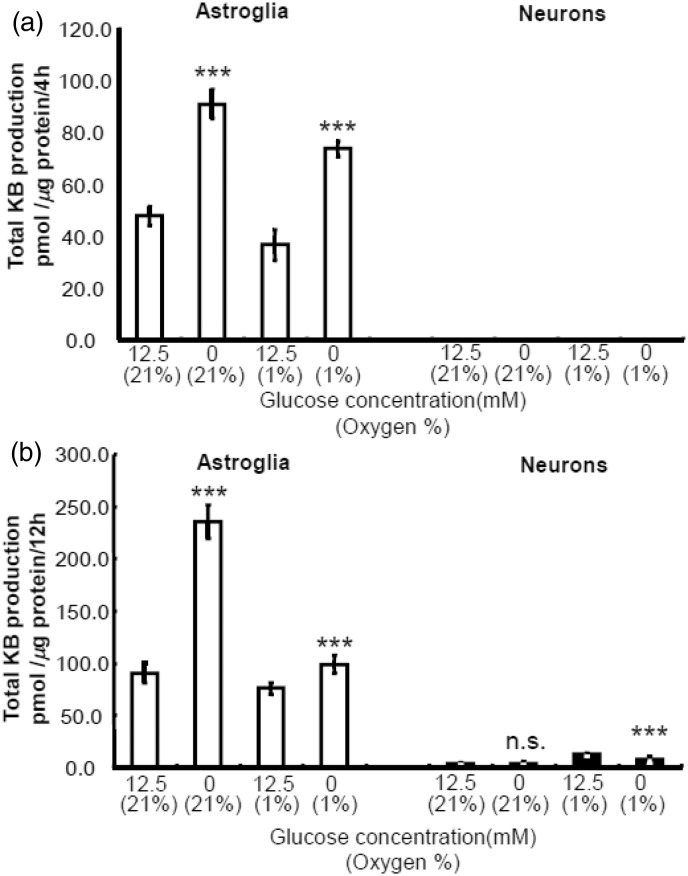
Effects of hypoxia with or without glucose-deprivation on ketogenesis from exogenous palmitic acid by neurons and astroglia. The effects of hypoxia (1% O_2_) with glucose (12.5 mmol/L) or without glucose for 4 hr (a) and 12 hr (b) on neuronal or astroglial total ketone body (KB) production were examined using DMEM containing no fetal bovine serum. Neither neurons nor astroglia tolerate hypoxia without glucose (i.e., oxygen-glucose deprivation, OGD) for 24 hr. Neuronal KB productions during 4 or 12 hr were very small and negligible. Hypoxia with glucose for 4 or12 hr did not elicit statistically significant increases in KB production by astroglia. Values are the mean ± SD (n = 6). n.s. = not significant. ***p < .001 (grouped *t* test).

To mimic clinical ischemic stroke, we examined the effect of combined insult of hypoxia without glucose (i.e., oxygen–glucose deprivation, OGD). For 24-hr hypoxia experiment, we used nutrient medium that contained 10% FBS to examine the effect of hypoxia *per se*. For OGD experiment, we removed FBS that contained low amount of glucose. We performed OGD experiment using two different conditions: DBSS without glucose (Figure 5(a) and (b)) or DMEM without glucose (Figure 6(a) and (b)). Because neither neurons nor astroglia tolerate OGD for 24 hr, we examined the effect of OGD for 4 and 12 hr. As shown in Figures 5 and 6, glucose deprivation alone induced marked elevation of KB synthesis in astroglia but not in neurons. Either 4 - or 12-hr incubation with glucose under hypoxia did not induce significant increases in KB production in astroglia. The different neuronal BHB production rates during hypoxia (Figures 4 and 5) might be ascribed to the assay solution (DBSS) that contains more restricted components (i.e., no amino acids or FBS, Figure 5) compared with assay media used for 24-hr hypoxia (i.e., nutrient media that may have provided supplemental substrates, Figure 4). In summary, the hypoglycemic component of OGD is the major factor involved in induction of a significant elevation in KB synthesis in astroglia after 4 and 12 hr, whereas KB synthesis is not altered by OGD.

### Neither Chemical Hypoxia Nor Glutamate Enhanced Total Ketogenesis in Neurons and Astroglia

**Figure 7.**
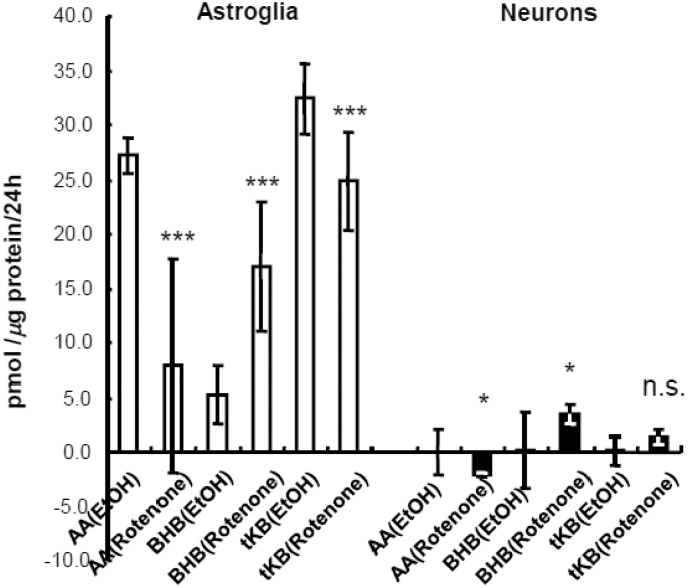
Effects of chemical hypoxia on ketogenesis by neurons and astroglia in the presence of exogenous palmitic acid and glucose. Production of acetoacetate (AA), β-hydroxybutyrate (BHB), and total ketone bodies (tKBs) in neurons (filled bars) and astroglia (blank bars), as determined using the cyclic thio-NADH method during 24 hr of exposure to rotenone or a vehicle with palmitic acid (PAL) and l-carnitine (LC). Glucose (2 mmol/L) was included in all assays. Rotenone reduced tKB production in astroglia compared with ethanol (EtOH)-treated control cells, but it did not affect neuronal tKB production. The BHB/AA ratio in both neurons and astroglia increased markedly in the presence of rotenone. Values are the mean ± SD (*n* = 6 for a and b). n.s. = not significant. *p < .05 (grouped *t* test).

Chemical hypoxia (rotenone, 1 µmol/L for 24 hr) in astroglia reduced AA production in the presence of PAL plus glucose but raised BHB formation and caused a 25% fall in total KB (i.e., sum of AA and BHB; see, cyclic thio-NADH method in the “Materials and Methods” section) production (Figure 7). BHB production was also enhanced in neurons. Thus, the BHB/AA ratio was markedly elevated in both types of cells, indicating that NADH/NAD^+^ increased as a result of the inhibition of the mitochondrial electron chain. Notably, astroglial ketogenesis in the presence of fatty acid plus substrates was lower in the presence of chemical hypoxia caused by rotenone (Figure 7) compared with oxygen deprivation (1% O_2_), that is, it was similar to that of the 4-hr interval of oxygen deprivation (Figures 5(a) and 6(a)) but about half that of the 12-hr interval (Figures 5(b) and 6(b)).

**Figure 8.**
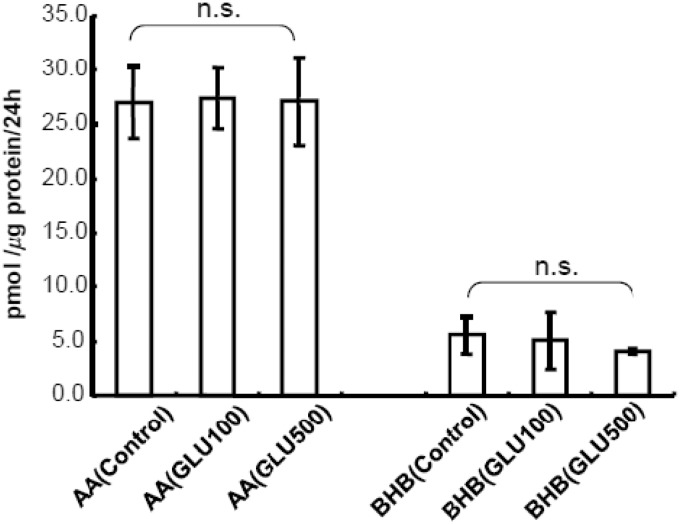
Effects of glutamate (GLU) on astroglial ketogenesis. Effects of GLU at different concentrations (100 and 500 µmol/L) on production of acetoacetate (AA) and β-hydroxybutyrate (BHB) in astroglia in the presence of glucose (2 mmol/L) were examined using the cyclic thio-NADH method during 24 hr of exposure to GLU (100 or 500 µmol/L). Values are the mean ± SD (*n* = 4). n.s. = not significant (AVOVA). **p < .01 (Dunnett test for multiple comparisons following ANOVA).

Furthermore, Guzmán and Blázquez (2001) mentioned the glutamate-induced enhancement of astroglial KB production as an unpublished observation. However, detailed data have not been presented. Hypoxic insults cause neuronal glutamate release (Nishizawa, 2001). Thus, whether glutamate *per se* induces astroglial KB production seems to be an important issue. We and others previously demonstrated that glutamate uptake through a Na^+^-dependent glutamate transporter enhances astroglial glucose utilization and lactate production (Pellerin and Magistretti, 1994; Takahashi et al., 1995). However, glutamate did not affect the astroglial production of AA or BHB (Figure 8) when assayed in the presence of glucose.

### Neuronal Oxidation of Lactate and BHB

**Figure 9.**
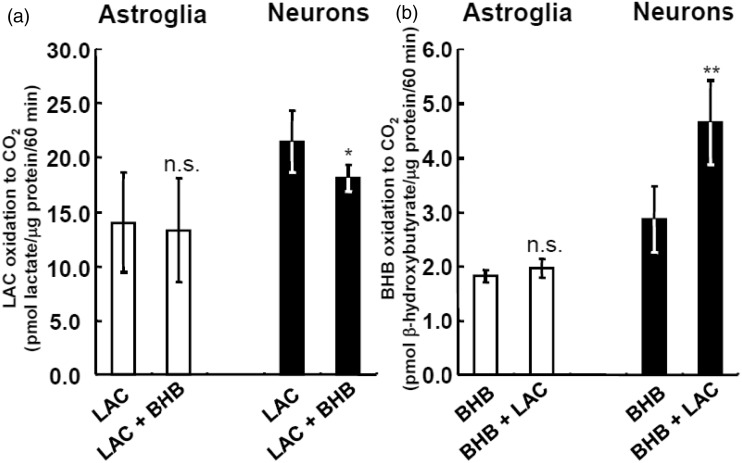
Neuronal oxidation of lactate (LAC) and β-hydroxybutyrate (BHB). (a) Effect of addition of BHB on LAC oxidation as determined using [U-^14^C]LAC in neurons (filled bars) and astroglia (blank bars). (b) Effect of addition of LAC on BHB oxidation as determined using [1-^14^C]BHB in neurons (filled bars) and astroglia (blank bars). Glucose was not included in the medium in these assays. Values are the mean ± SD (*n* = 4). n.s. = not significant. *p < .05; **p < .01 (grouped *t* test)**.**

In neurons, LAC oxidation was reduced by the addition of BHB (Figure 9(a), right), and BHB oxidation was augmented by the addition of LAC (Figure 9(b), right). Neither LAC nor BHB oxidation was affected by the addition of BHB or LAC in astroglia (Figures 9(a) and (b)). Notably, BHB oxidation may supplement lactate as fuel, but its overall contribution is relatively small. The role of lactate and BHB as energy substrates for astroglial oxidative metabolism is another important issue to be solved (McKenna, 2012).

### Effects of 1% Hypoxia (24 hr) on Oxidative Metabolism of Lactate, Pyruvate, or BHB in Neurons

**Figure 10.**
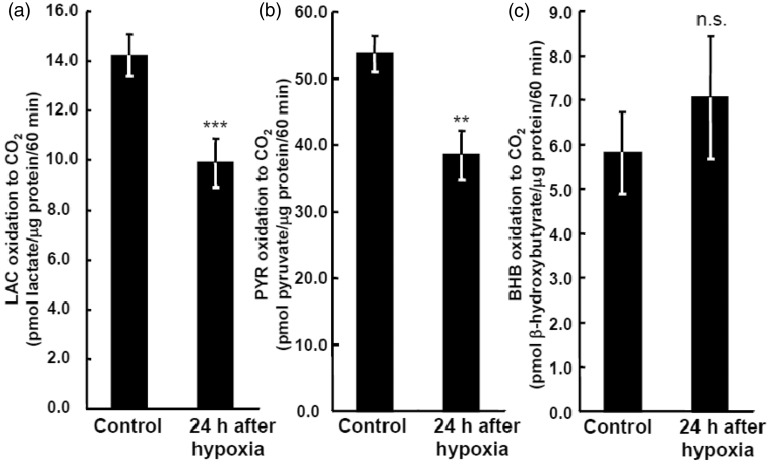
Hypoxia/reoxygenation-induced alteration of neuronal oxidation of lactate (LAC), pyruvate (PYR), and β-hydroxybutyrate (BHB). (a) Effects of hypoxia (1% O_2_ for 24 hr) on [U-^14^C]LAC in neurons. (b) Effects of hypoxia (1% O_2_ for 24 hr) on [1-^14^C] PYR in neurons. (c) Effects of hypoxia (1% O_2_ for 24 hr) on [1-^14^C]BHB in neurons. Glucose was not included in the medium in these assays, which were carried out in 21% oxygen after the 24-hr hypoxic interval. Values are the mean ± SD (*n* = 4). n.s. = not significant. **p < .01; ***p < .001 (grouped *t* test).

The neuronal utilization (oxidative metabolism) of LAC (Figure 10(a)) and PYR (Figure 10(b)) was significantly reduced when assayed under normoxic conditions after hypoxia (24 hr), while BHB oxidation (Figure 10(c)) was preserved (no statistically significant decrease, grouped *t* test). In control neuronal cultures, pyruvate was oxidized 3.8-fold faster than lactate and 8.8 times better than BHB (Figure 10), indicating that pyruvate is the best neuronal substrate and that shuttling of NADH equivalents derived from lactate into mitochondria may limit lactate oxidation compared with that of pyruvate.

## Discussion

The present study demonstrated that both cultured astroglia and neurons produce KBs from long-chain fatty acids, that is, PAL, while fatty acid oxidation through the TCA cycle was negligible. Regarding glucose oxidation, neurons exhibit a much higher rate of glucose oxidation, whereas astroglia possess a lower capacity for glucose oxidation irrespective of the higher glucose utilization, resulting in a large amount of lactate production (Izawa et al., 2009). Both neurons and astroglia produce lactate under hypoxia, and the present study demonstrated that neurons produced BHB probably from endogenous lipids or other compounds in the medium. In contrast, astroglial KB production was exogenous fatty acid derived but not glucose derived. AMPK activation enhanced astroglial KB production through ACC phosphorylation, and hypoxia may also enhance KB production through the same mechanism, as reported by Guzmán and Blázquez (2001).

Although the function of the adult brain is completely dependent on glucose oxidation for ATP production, suckling baby mainly utilizes KBs instead of glucose. Even the adult brain shifts its energy source from glucose to KBs under conditions of starvation or insulin resistance associated with diabetes mellitus, but increased KB utilization is associated with upregulation of monocarboxylate transporters in the BBB to facilitate greater KB uptake into brain (Pan et al., 2000, 2001, 2002).

An important finding of the present study is that both astroglia and neurons utilize fatty acid to produce KBs instead of utilizing it as a TCA cycle substrate. Astroglia produced more KBs than neurons, as reported by Guzmán and Blázquez (2001). They showed that AICAR (an AMPK activator) stimulates astroglial KB production by phosphorylating ACC. Because phosphorylation inhibits ACC activity, the level of malonyl-CoA, which is a main physiological inhibitor of CPT-I (a rate-limiting enzyme of fatty acid metabolism), is reduced.

The present study only partly confirmed their observation, as cilostazol, which also cause phosphorylation of ACC, failed to stimulate KB production, suggesting that other mechanisms may be involved. We previously reported that a clinically relevant concentration of cilostazol (3 µmol/L) did, indeed, alter glucose metabolism in cultured astroglia through PKA (Takahashi et al., 2011) Therefore, cilostazol at this concentration should have activated PKA, leading to the augmentation of KB production. The lack of an effect of cilostazol on fatty acid metabolism in cultured astroglia remains to be explored.

Another activator of AMPK, metformin, is clinically used for the treatment of diabetes mellitus. The lower concentration (up to 100 µmol/L) of metformin used in the present study was selected based on the estimated concentration in the brain of patients as determined by calculating the peak blood concentration of metformin assuming a BBB permeability of approximately 10% of the concentration in the blood. At this concentration, metformin did not induce KB production in astroglia. In contrast, the higher concentration (1,000 µmol/L) did indeed induce KB production. Interestingly, however, Fulgencio et al. (2001) reported that metformin (50–500 µmol/L) suppressed ketogenesis and ketogenic gene expression in freshly isolated hepatocytes. The reason for the contradictory results between hepatocytes and cultured astroglia remains to be determined.

A suggestion made a decade ago that metformin reduces glucose synthesis through the activation of AMPK has recently been challenged by genetic loss-of-function experiments (Foretz et al., 2010). According to Miller et al. (2013), metformin antagonizes the action of glucagon, thus reducing the fasting glucose levels. In mouse hepatocytes, metformin leads to the accumulation of AMP and related nucleotides, which inhibit adenylate cyclase, reduce the levels of cAMP and PKA activity, abrogate the phosphorylation of critical protein targets of PKA, and block glucagon-dependent glucose output from hepatocytes. In the present study, cilostazol activated PKA but did not alter KB production, as shown earlier. Whether the mechanism by which a higher concentration of metformin induces astroglial ketogenesis involves this novel pathway remains uncertain.

A novel finding of the present study is that neurons under hypoxia produce substantial amounts of KBs. Moreover, this hypoxia-induced KB production by neurons was not exogenous PAL dependent. These results suggest that acetyl-CoA from other sources, which normally enters the TCA cycle, is instead used for KB production under hypoxic conditions. However, astroglia KB production under hypoxia disappeared with the elimination of PAL. As KBs are known to play neuroprotective roles under ischemia, KBs derived from both neurons and astrocytes might have important roles in the brain *in vivo*. In particular, the roles of astroglia seem to be important, as they produce KBs directly from exogenous fatty acids.

Guzmán and Blázquez (2001) reported that chemical hypoxia also enhanced neuronal KB production. In the present study, however, somewhat different results were obtained. The total KB (AA and BHB) production in the neurons did not increase with the addition of rotenone, a mitochondrial complex I inhibitor. Furthermore, rotenone actually reduced the total KB production in astroglia. Both types of cells showed an increase in the ratio of BHB/AA, indicating that NADH/NAD^+^ associated with rotenone-induced mitochondrial dysfunction. Moreover, glucose deprivation seemed to be more a potent activator of astroglial ketogenesis. Astroglial energy production seems to be more dependent on glycolytic metabolism rather than oxidative metabolism of glucose (Abe et al., 2006; Takahashi et al., 2012b). The results of the present study are in accordance with recent findings by Taïb et al. (2013), who found that hypothalamic astroglia responds to hypoglycemia by generating KBs *in vivo*.

The fate of KBs produced by either neurons or astroglia seems to be important because reoxygenation after hypoxia facilitates ROS production from damaged mitochondria, leading to PDHC impairment. *In vivo* studies showed that PDHC activity is enhanced during transient ischemia and then decreases after reperfusion (Cardell et al., 1989; Fukuchi et al., 1998). Thus, even though lactate accumulates during hypoxia, it may not be readily available as an energy source after reoxygenation. The present study clearly demonstrated that lactate and pyruvate oxidation decreased in neurons after hypoxia/reoxygenation, while BHB oxidation was preserved. Therefore, it is reasonable to speculate that KBs produced by astroglia or neurons, or both cell types, can fuel the neuronal energy state after hypoxia/reoxygenation. Whether KBs produced during ischemia support astroglial oxidative metabolism (McKenna, 2012) after reoxygenation is subject of the future study.

### Conclusion

Astroglia and neurons responded to hypoxia by enhancing KB production, and KBs produced by astroglia or neurons or both might be used as a neuronal energy substrate. The activation of astroglial ketogenesis through activated AMPK might reduce ischemic cell damage.
